# Supplementary material for: Supplementation of Enriched Polyunsaturated Fatty Acids and CLA Cheese on High Fat Diet: Effects on Lipid Metabolism and Fat Profile
Source: Foods. 2022 Jan 29;11(3):398. doi: 10.3390/foods11030398 (PMC8834222; doi:10.3390/foods11030398)
Supplement: Supplementary file 1 [file foods-11-00398-s001.zip › foods-1532555-supplementary.pdf]

Supplementary File - for Online Publication Only

Supplementation of enriched polyunsaturated fatty acids and CLA cheese on High Fat Diet: effects on lipid metabolism and fat profile.

M. Tognocchi, M. Conte, L. Testai, M. Martucci, A. Serra, S. Salvioli, V. Calderone, M. Mele, and G. Conte

Supplementary Table S1. Genes analyzed in the work.

**Genes involved in Lipid metabolism**	
PLIN1 (Perilipin 1)	Perilipin 1 is a protein that coats lipid droplets in adipocytes. the fat-storing cells in adipose tissue. In fact. PLIN1 is greatly expressed in white adipocytes. It controls adipocyte lipid metabolism. by the regulation of basal and hormonally stimulated lipolysis and also the formation of large lipid droplets which implies an increase in the synthesis of triglycerides.
PLIN2 (Perilipin 2)	Perilipin 2 is ubiquitously expressed and represents the major perilipin associated with the drop of fat. This protein is involved in the regulation of the lipolytic process
PLIN3 (Perilipin 3)	Perilipin 3 role is not completely defined. but may be involved in the regulation of lipolitic activity.
PLIN4 (Perilipin 4)	Perilipin 4 is associated with lipid drop development.
PLIN5 (Perilipin 5)	Perilipin 5 plays a key role in the regulation of lipolysis of fat droplets. Thanks to this control. it controls the storage of triglycerides and their lipolysis.
SREBP-1 (Sterol Regulator Element Binding Protein)	SREBP-1 plays a key role in the induction of lipogenesis
ACACA (Acetyl-CoA carboxylase alpha)	ACACA synthesized acetyl-CoA carboxylase 1. a cytosolic enzyme that induces the carboxylation of acetyl-CoA into Malonyl-CoA. which represents the first step in the biosynthesis of fatty acids.
ACACB (Acetyl-CoA carboxylase beta)	ACACB synthesized acetyl-CoA carboxylase 2. a mitochondrial enzyme that catalyzes the carboxylation of acetyl-CoA into malonyl-CoA and plays an important role in the metabolism of fatty acid synthesis.
FASN (Fatty Acid Synthase)	FASN synthesized Fatty Acid Synthase. which catalyzed the elongation of acylic chain during fatty acid synthesis.
SCD (Steroyl CoA Desaturse)	SCD synthesized Stearoyl CoA Desaturase. which is involved in the cis-9 desaturation of fatty acids.
GPAM (Glycerol-3-phosphate acyltransferase)	GPAM catalyzes the initial and committing step in glycerolipid biosynthesis. is predicted to play a pivotal role in the regulation of cellular triacylglycerol and phospholipid levels.
AGPAT2 (Acetilglicerol-3fosfato O-acetiltransferasi)	AGPAT2 synthesized Acetilglicerol-3fosfato O-acetiltransferasi. which is involved in the second step of triglycerides synthesis
DGAT1 (Diacylglycerol O-acyltransferase 1)	Diacylglycerol O-acyltransferase 1 catalyzes the last step in the synthesis of triglycerides. using diaglycerol and acetyl-CoA as substrate. It could carry out the assembly of the VLDLs. In the liver it plays an important role in the exogenous esterification of fatty acids to glycerol.
DGAT2 (Diacylglycerol O-acyltransferase 2)	Diacyglycerol O-acetyltransferase 2 catalyzes the last step of the synthesis of triglycerides using diaglycerol and acetyl-CoA as substrate. It is responsible for the synthesis of endogenous triglycerides.
LPIN1 (phosphatidate phosphatase)	Phosphatidine phosphatase catalyzes the conversion of phosphatidic acid into diglycerol during the formation of triglyceride. It is also a co-activator of nuclear transcription and regulates the pathway of the expression of lipid metabolism genes. It is also involved in the differentiation of adipocytes.
ELOVL2 (Fatty acid elongase 2)	Catalyzes the first step of the four reactions that make up the Very Long Chain Fatty Acids elongation cycle.

ACLY (ATP Citrate Synthase)	ATP citrate synthetase catalyzes the splitting of citrate into oxaloacetate and acetyl-CoA. the latter used to synthesize cholesterol and fatty acids.
ETNK1 (Ethanolamine kinase 1)	Ethanolamine kinase 1 is involved in the Kennedy pathway. which regulates the first step of the synthesis of phosphatidylethalonamine. This cytosolic enzyme is specific for ethanolamine and also shows negligible kinase activity on choline.
PCYT2 (Ethanolamine-phosphate cytidylyltransferase	Ethanolamine-phosphatase cytidyltrasferase plays an important role in the biosynthesis of phospholipid-phosphaditylethalonamine. This enzyme is also involved in the Kennedy pathway.
CEPT-1 (Choline/ethanolaminephosphotransferase 1)	It catalyzes both the biosynthesis of phosphatidylcholine and phosphatidylethalonamine starting from choline ethanolamine phosphotransesferase1. It involves a dependent protein in the transport of phospholipids and distributes the phosphotydilcholine on the luminal surface. It also has a better colinphosphotransferase activity than the activity of ethanolamine phosphotransferase. It is involved in the Kennedy pathway.
PISD (Phosphatidylserine Decarboxylase)	It catalyzes the formation of phosphatidylethanolamine from phosphatidylserine. It plays a central role in phospholipid metabolism within the phosphatidylserine organelles. It intervenes in the Kennedy pathway.
CERK (Ceramide kinase)	Ceramide kinase enhances cell survival and proliferation. It is the promoter of DNA synthetase in fibroblasts. it also prevents apoptosis. it also interacts by blocking the functionality of the spingomilesin acid. It has recently been seen that it stimulates the proliferation of myoblasts and that it induces the phosphorylation of glucose-synthase. It intervenes in the synthesis of ceramides.
CERS6 (Ceramide synthase 6)	Ceramide synthase catalyzes the formation of sphinganine ceramide and the substrates of acetyl-CoA. With a high selectivity towards palmothyl-CoA as an acyl donor.
PCK1 (Phosphoenolpyruvate carboxykinase 1)	Phosphoenolpyruvate carboxylase is involved in the production of oxaloacetate into phosphoenolpyruvate and through the gluconogenic pathway that forms glucose.
PRKAA1 (Protein Kinase AMP-Activated Catalytic Subunit Alpha 1)	It catalyzes the AMP-activating protein kinase subunit. that plays a key role in the regulation of cellular lipid metabolism.
ATGL (or PNPLA2 - Patatin-like phospholipase domain-containing protein 2)	It catalyzes the initial step of hydrolysis of triglycerides in adipocytes and non-adipocytes lipid droplets. It coordinates the lipolytic cascade. regulates the size of adiposomes and could be involved in the degradation of adiposomes. It also has an important role in energy homeostasis.
CPT1B (Carnitine O-palmitoyltransferase 1)	Carnetin O-palmitoltransferase1 is involved in the beta oxidation pathway of fatty acids.
ATGL (Adipose triglycerides lipase)	Adipose triglycerides lipase catalyzed the initial step of triglyceride hydrolysis. It can play an important role in energy homeostasis and in the response to hunger. by promoting the hydrolysis of triglycerides.
HSL (Hormone Sensitive Lipase)	It hydrolyzes triglycerides to free fatty acids and converts cholesterol esters into free sterol. for the production of the steroid hormone.
LIPE (Lipase E)	Lipase E with the function of hydrolyzing other fatty acids from triacylglycerol molecules. releasing fatty acids and diglycerides or fatty acids from diglyceride molecules. it also frees fatty acids from monoglycerides.
**Genes involved in inflammation and cellular cycle**	
NFATC2 (Nuclear Factor Of Activated T Cells 2)	Nuclear factor of activation of cytoplasmic cells plays an important role in inducing the expression of cytokine genes in T cells. in particular IL-2. IL-3. IL-4 and TNF-alpha. Involved in the inflammatory response.
FGF21 (Fibroblast Growth Factor 21)	FGF21 is specifically induced by mitochondrial 3-hydroxy-3-methylglutaryl-CoA synthase 2 (HMGCS2) activity. The oxidized form of ketone bodies (acetoacetate) in a cultured medium also induced FGF21. possibly via a sirtuin 1 (SIRT1)-dependent mechanism.[14] HMGCS2 activity has also been shown to be increased by deacetylation of lysines 310. 447. and 473 via SIRT3 in the mitochondria.

PRKCQ (Protein kinase C theta type)	Phospholipid- and diglyceride-dependent kinase regulates the functions in T-cell receptor (TCR) signals. including their activation. proliferation and differentiation.
TRP53 (Transformation related protein 53)	Encodes a cancer cell antigen. calcium dependent. triggering cell proliferation.
P21 (Cyclin-dependent kinase inhibitor 1)	It is a potent inhibitor of cyclin-dependent kinases. It regulates the progression of the cell cycle between the G1 and S phase. Its expression is strongly controlled by P53.
IL1B	IL-1β is a member of the interleukin 1 family of cytokines. This cytokine is produced by activated macrophages as a proprotein. which is proteolytically processed to its active form by caspase 1 (CASP1/ICE). This cytokine is an important mediator of the inflammatory response and is involved in a variety of cellular activities. including cell proliferation. differentiation. and apoptosis.
GDF15 (Growth Differentiation Factor 15)	It regulates food intake. energy consumption and body weight. in response to metabolic and toxin-induced stress.
**Genes involved in mitochondrial metabolism**	
PGC1A (Peroxisome proliferator-activated receptor gamma coactivator 1-alpha)	It is a transcriptional coactivator for steroid and nuclear receptors. It coordinates the expression of the genes involved with glucose and the metabolism of fatty acids.
OPA1 (Dynamin-like 120 kDa protein)	It regulates the balance between fusion and mitochondrial fission. It plays an important role in the release of cytochrome during apoptosis. Regulates the maintenance of the mitochondrial genome.
MFN1 (Mitofusin 1)	Mitofusin1 is an active GTPase of the mitochondrial outer membrane. which regulates mitochondrial fusion.
DRP1 (Dynamin-related Protein)	It is involved in the mitochondrial division and the peroximal division.
UCP. UCP2. UCP3 (Uncoupling protein)	The Uncoupling proteins are transporter that creates a proton loss of the mitochondrial membrane.
NDUFS3 (NADH:Ubiquinone Oxidoreductase Core Subunit S3)	It is the complex I of the mitochondrial respiratory chain. This complex is involved in the transfer of electrons from NADH to the respiratory chain.
UQCR10 (Ubiquinol-Cytochrome C Reductase. Complex III Subunit X)	It is the complex III of the mitochondrial respiratory chain.
COX10	It is the complex IV of the mitochondrial respiratory chain.
ATP5G1	It is the complex V of the mitochondrial respiratory chain.
KLOTOB	

Supplementary Table S2. Effect of diet on Fatty Acid profile (g/100g of total lipids) of liver.

	CON	CHE	ENR	SE	P-value
C8:0	0.09^{b}	0.12^{b}	0.51^{a}	0.01	*
C10:0	0.33	0.22	0.38	0.08	ns
C12:0	0.08^{b}	0.32^{a}	0.45^{a}	0.07	*
C13iso	0.19^{b}	0.09^{b}	0.47^{a}	0.02	*
C13ante	0.03	0.11	0.04	0.02	ns
C13:0	0.02	0.10	0.03	0.01	ns
C14:0	0.83^{B}	2.69^{A}	2.87^{A}	0.25	***
C15iso	0.10^{C}	0.31^{B}	0.45^{A}	0.10	***
C15ante	0.06^{b}	0.22^{a}	0.23^{a}	0.05	*
C14:1c9	0.09^{b}	0.15^{a}	0.19^{a}	0.02	*
C15:0	0.22^{C}	0.63^{B}	0.72^{A}	0.04	***
C16iso	0.09^{b}	0.29^{a}	0.20^{a}	0.04	*
C16:0	22.29^{b}	27.06^{a}	25.22^{a}	0.80	**
C17:0	0.08^{b}	0.29^{a}	0.25^{a}	0.04	*
C16:1c7	1.06^{a}	0.58^{b}	0.77^{b}	0.09	*
C16:1c9	4.26^{a}	2.56^{c}	3.18^{b}	0.31	*
C17:0	0.24^{C}	0.88^{A}	0.62^{B}	0.06	***
C17:1c9	0.14^{a}	0.29^{c}	0.36^{b}	0.04	**
C18:0	3.44^{C}	7.60^{A}	6.37^{B}	0.50	***
C18:1t6-8	0.01^{c}	0.30^{b}	0.50^{a}	0.07	*
C18:1t9	0.01^{c}	0.24^{b}	0.75^{a}	0.12	**
C18:1t10	0.00^{c}	0.50^{b}	0.85^{a}	0.12	**
C18:1t11	0.00^{C}	0.99^{B}	1.55^{A}	0.15	***
C18:1c9	26.71	29.31	30.93	1.89	ns
C18:1c11	1.76^{A}	0.93^{B}	0.76^{B}	0.11	***
C18:1c12	0.00^{C}	0.27^{B}	0.42^{A}	0.06	**
C18:2cc	29.29^{A}	8.93^{B}	9.16^{B}	0.95	***
C20:0	0.16	0.55	0.58	0.15	ns
C18:3n6	0.48	0.25	0.37	0.07	ns
C20:1c11	0.57	0.66	0.86	0.13	ns
C18:3n3	1.04	0.89	0.93	0.13	ns
C21:0	0.05^{b}	0.24^{a}	0.23^{a}	0.04	**
C18:4n3	0.12	0.20	0.34	0.07	ns
C20:2n6	0.34	0.35	0.30	0.13	ns
C22:0	0.09^{b}	0.31^{a}	0.41^{a}	0.08	*
C20:3n6	0.18	0.41	0.47	0.12	ns
C20:3n3	0.06^{C}	0.13^{B}	0.52^{A}	0.06	***
C22:1c13	0.07^{b}	0.20^{a}	0.06^{b}	0.03	*
C20:4n6	1.68	2.08	1.50	0.26	ns
C23:0	0.15	0.20	0.19	0.05	ns
C22:2	0.00^{c}	0.16^{b}	0.21^{a}	0.04	*
C23:1c14	0.27^{A}	0.00^{C}	0.08^{C}	0.01	***
C20:5n3	1.09^{a}	0.45^{b}	0.48^{b}	0.12	**
C22:3n3	0.35^{A}	0.08^{B}	0.00^{C}	0.03	***
C24:1c15	0.26^{a}	0.00^{c}	0.07^{b}	0.04	**
C22:4n6	0.12^{c}	0.62^{a}	0.38^{b}	0.11	*
C22:5n6	0.15	0.41	0.35	0.12	ns
C22:5n3	0.17^{c}	0.77^{a}	0.63^{b}	0.14	*
C22:6n3	1.15^{C}	2.59^{A}	1.83^{B}	0.17	***

SE= Standard Error; a.b.c (differences on the row $P<0.05$); A.B.C (differences on the row $P<0.01$); ns: no significant; *: $0.01 <$ P-value ≤ 0.05; **: $0.001 <$ P-value ≤ 0.01; ***: P-value ≤ 0.001.

Supplementary Table S3. Effect of diet on Fatty Acid profile (g/100g of total lipids) of brain.

	CON	CHE	ENR	SE	P-value
C8:0	0.68	0.84	0.61	0.18	ns
C10:0	0.94	1.34	1.03	0.28	ns
C12:0	0.45	1.06	0.93	0.22	ns
C13iso	0.14^{c}	0.60^{a}	0.30^{b}	0.10	*
C13ante	0.15	0.25	0.25	0.09	ns
C13:0	0.21	0.20	0.17	0.07	ns
C14:0	2.48	2.56	2.68	0.41	ns
C15iso	0.71	1.49	1.02	0.27	ns
C15ante	0.26	0.65	0.66	0.22	ns
C14:1c9	0.22^{C}	0.40^{B}	1.27^{A}	0.13	***
C15:0	0.48	0.63	0.65	0.13	ns
C16iso	0.39	0.59	0.66	0.19	ns
C16:0	18.96	20.60	17.49	1.29	ns
C17:0	0.26	0.36	0.29	0.09	ns
C16:1c7	0.23^{c}	0.42^{b}	1.00^{a}	0.11	**
C16:1c9	0.70	1.01	1.29	0.24	ns
C17:0	0.71	0.99	0.98	0.16	ns
C17:1c9	0.40	0.89	0.70	0.23	ns
C18:0	13.92	14.72	11.70	1.05	ns
C18:1t6-8	0.11	0.27	0.29	0.09	ns
C18:1t9	0.12	0.23	0.25	0.03	ns
C18:1t10	0.41	0.38	0.34	0.17	ns
C18:1t11	0.79	1.08	1.13	0.31	ns
C18:1c9	22.91	22.64	19.27	2.09	ns
C18:1c11	2.14^{a}	2.38^{a}	1.19^{b}	0.28	*
C18:1c12	0.20	0.16	0.29	0.04	ns
C18:2cc	6.38	4.60	5.64	1.06	ns
C20:0	1.13	1.20	1.35	0.29	ns
C18:3n6	0.32	0.46	0.69	0.18	ns
C20:1c11	1.36	0.98	1.30	0.32	ns
C18:3n3	0.83	1.45	1.44	0.34	ns
C21:0	0.73	1.03	1.37	0.16	ns
C18:4n3	0.40	0.75	0.72	0.14	ns
C20:2n6	0.49	0.26	0.81	0.20	ns
C22:0	0.57^{b}	0.37^{b}	1.07^{a}	0.19	*
C20:3n6	0.36^{c}	0.72^{b}	1.16^{a}	0.17	*
C20:3n3	0.10^{b}	0.74^{a}	0.65^{a}	0.15	*
C22:1c13	0.12	0.41	0.26	0.09	ns
C20:4n6	4.94	5.44	3.41	0.65	ns
C23:0	0.47^{c}	0.78^{b}	1.04^{a}	0.14	*
C22:2	0.19^{C}	0.31^{B}	1.33^{A}	0.11	***
C23:1c14	0.00^{C}	0.04^{B}	0.22^{A}	0.02	***
C20:5n3	0.61^{b}	0.46^{b}	1.10^{a}	0.16	*
C22:3n3	0.03	0.01	0.14	0.04	ns
C24:1c15	0.13	0.00	0.19	0.05	ns
C22:4n6	0.68	1.20	0.84	0.19	ns
C22:5n6	0.45	0.57	0.64	0.12	ns
C22:5n3	0.66	0.96	0.93	0.33	ns
C22:6n3	1.58	2.13	1.59	0.58	ns

SE= Standard Error; a.b.c (differences on the row P<0.05); A.B.C (differences on the row P<0.01); ns: no significant; *: 0.01 < P-value ≤ 0.05; **: 0.001 < P-value ≤ 0.01; ***: P-value ≤ 0.001.

Supplementary Table S4. Effect of diet on Fatty Acid profile (g/100g of total lipids) of skeletal muscle.

	CON	CHE	ENR	SE	P-value
C8:0	0,36^{b}	1,63^{a}	1,71^{a}	0,24	**
C10:0	0,71^{b}	1,94^{a}	1,14^{b}	0,22	*
C12:0	0,56^{b}	1,18^{a}	0,68^{b}	0,12	*
C13iso	0,09^{c}	0,22^{b}	0,40^{a}	0,06	*
C13ante	0,02	0,06	0,09	0,02	ns
C13:0	0,05^{c}	0,15^{b}	0,31^{a}	0,05	*
C14:0	3,46^{b}	4,73^{a}	3,26^{b}	0,43	*
C15iso	0,21^{B}	4,40^{A}	4,39^{A}	0,53	***
C15ante	0,18^{c}	0,56^{b}	0,78^{a}	0,14	*
C14:1c9	0,32	0,33	0,56	0,08	ns
C15:0	0,48^{c}	0,80^{b}	1,08^{a}	0,14	*
C16iso	0,19^{c}	0,30^{b}	0,45^{a}	0,05	*
C16:0	22,07^{b}	27,06^{a}	21,08^{b}	1,81	*
C17:0	0,23	0,17	0,44	0,11	ns
C16:1c7	0,68^{b}	0,75^{b}	1,22^{a}	0,12	*
C16:1c9	8,39^{a}	3,78^{b}	3,93^{b}	0,68	**
C17:0	0,63^{c}	0,98^{a}	0,78^{b}	0,06	*
C17:1c9	0,21	0,26	0,30	0,03	ns
C18:0	10,51	17,00	15,50	2,15	ns
C18:1t6-8	0,29	0,41	0,48	0,14	ns
C18:1t9	0,08^{c}	0,20^{b}	0,60^{a}	0,13	*
C18:1t10	0,05^{c}	0,27^{b}	0,52^{a}	0,07	**
C18:1t11	0,54^{b}	1,14^{a}	1,45^{a}	0,20	*
C18:1c9	25,42^{a}	17,67^{b}	18,50^{b}	1,34	**
C18:1c11	1,87^{A}	0,82^{C}	1,01^{B}	0,10	***
C18:1c12	0,00^{C}	0,05^{B}	0,33^{A}	0,03	***
C18:2cc	16,55^{A}	5,48^{B}	5,05^{B}	1,27	***
C20:0	0,49^{B}	0,99^{A}	0,95^{A}	0,05	***
C18:3n6	0,10^{c}	0,38^{b}	0,82^{a}	0,15	**
C20:1c11	0,30^{C}	1,66^{B}	1,96^{A}	0,27	***
C18:3n3	0,68	0,50	1,20	0,32	ns
C21:0	0,57	0,87	0,87	0,14	ns
C18:4n3	0,10^{C}	0,20^{B}	0,57^{A}	0,06	***
C20:2n6	0,19^{b}	0,17^{b}	0,84^{a}	0,16	*
C22:0	0,15^{c}	0,24^{b}	0,86^{a}	0,14	*
C20:3n6	0,20^{c}	1,41^{b}	1,87^{a}	0,24	**
C20:3n3	0,08^{c}	0,20^{b}	0,29^{a}	0,03	**
C22:1c13	0,14	0,18	0,19	0,06	ns
C20:4n6	0,98	1,49	1,28	0,39	ns
C23:0	0,14^{b}	0,14^{b}	0,62^{a}	0,07	**
C22:2	0,01^{C}	0,08^{B}	0,21^{A}	0,02	***
C23:1c14	0,09	0,08	0,25	0,05	ns
C20:5n3	0,29^{b}	0,21^{b}	0,67^{a}	0,12	*
C22:3n3	0,15^{b}	0,00^{c}	0,28^{a}	0,05	*
C24:1c15	0,14^{b}	0,03^{c}	0,36^{a}	0,09	*
C22:4n6	0,25^{c}	0,30^{b}	0,66^{a}	0,15	*
C22:5n6	0,10^{c}	0,22^{b}	0,92^{a}	0,19	*
C22:5n3	0,09	0,33	1,19	0,44	ns
C22:6n3	0,19^{c}	0,51^{b}	1,10^{a}	0,23	*

SE= Standard Error; a.b.c (differences on the row P<0.05); A.B.C (differences on the row P<0.01); ns: no significant; *: 0.01 < P-value ≤ 0.05; **: 0.001 < P-value ≤ 0.01; ***: P-value ≤ 0.001.

Supplementary Table S5. Effect of diet on Fatty Acid profile (g/100g of total lipids) of adipose tissue.

	CON	CHE	ENR	SE	P-value
C8:0	0.07^{B}	0.31^{A}	0.39^{A}	0.12	*
C10:0	0.07^{C}	0.41^{A}	0.33^{B}	0.07	**
C12:0	0.01^{B}	0.69^{A}	0.53^{A}	0.07	*
C13iso	0.01	0.02	0.04	0.01	ns
C13ante	0.00	0.00	0.00	-	ne
C13:0	0.01	0.01	0.01	0.01	ns
C14:0	0.72^{B}	3.99^{A}	3.51^{A}	0.25	***
C15iso	0.31^{b}	0.44^{b}	0.86^{a}	0.09	*
C15ante	0.03^{b}	0.16^{a}	0.17^{a}	0.05	*
C14:1c9	0.04^{b}	0.27^{a}	0.22^{a}	0.02	*
C15:0	0.16^{B}	0.76^{A}	0.70^{A}	0.04	***
C16iso	0.12^{b}	0.25^{a}	0.23^{a}	0.04	*
C16:0	18.18	19.22	18.84	0.81	ns
C17:0	0.01^{C}	0.79^{B}	0.96^{A}	0.04	**
C16:1c7	5.07	6.41	5.85	0.09	ns
C16:1c9	0.29^{b}	0.52^{a}	0.50^{a}	0.31	*
C17:0	0.23^{B}	0.62^{A}	0.54^{A}	0.06	***
C17:1c9	1.70^{B}	2.50^{A}	2.79^{A}	0.04	**
C18:0	2.47^{b}	3.47^{ab}	4.18^{a}	0.49	*
C18:1t6-8	0.01^{b}	0.18^{ab}	0.33^{a}	0.17	*
C18:1t9	0.00^{B}	0.27^{B}	0.51^{A}	0.12	**
C18:1t10	0.01^{C}	0.32^{B}	1.02^{A}	0.11	**
C18:1t11	0.00^{C}	0.71^{B}	2.16^{A}	0.15	***
C18:1c9	33.84^{C}	36.54^{B}	40.06^{A}	1.88	**
C18:1c11	0.10^{B}	0.19^{B}	0.60^{A}	0.10	***
C18:1c12	0.01^{C}	0.15^{B}	0.55^{A}	0.05	***
C18:2cc	31.14^{A}	10.47^{B}	9.45^{B}	0.95	***
C20:0	0.09	0.15	0.16	0.15	ns
C18:3n6	0.09^{B}	0.05^{B}	0.40^{A}	0.07	**
C20:1c11	0.74	0.53	0.79	0.13	ns
C18:3n3	1.05	0.59	0.60	0.04	ns
C21:0	0.02^{B}	0.02^{B}	0.05^{A}	0.00	**
C18:4n3	0.01^{b}	0.02^{b}	0.06^{a}	0.12	*
C20:2n6	0.13	0.06	0.07	0.07	ns
C22:0	0.01^{B}	0.05^{A}	0.07^{A}	0.01	*
C20:3n6	0.28	0.18	0.32	0.06	ns
C20:3n3	0.06	0.05	0.04	0.03	ns
C22:1c13	0.31^{a}	0.12^{b}	0.07^{b}	0.26	*
C20:4n6	0.04	0.15	0.19	0.05	ns
C23:0	0.00	0.09	0.08	0.04	ns
C22:2	0.00^{c}	0.01^{b}	0.02^{a}	0.00	*
C23:1c14	0.00	0.00	0.00	-	ne
C20:5n3	0.01	0.03	0.02	0.00	ns
C22:3n3	0.00	0.00	0.00	-	ne
C24:1c15	0.00	0.00	0.00	-	ne
C22:4n6	0.13	0.02	0.05	0.01	ns
C22:5n6	0.05	0.05	0.03	0.00	ns
C22:5n3	0.05	0.09	0.04	0.00	ns
C22:6n3	0.06	0.08	0.02	0.01	ns

SE= Standard Error; a.b.c (differences on the row $P<0.05$); A.B.C (differences on the row $P<0.01$); ns: no significant; ne: no estimable *: 0.01 < P-value ≤ 0.05; **: 0.001 < P-value ≤ 0.01; ***: P-value ≤ 0.001.
